# Evaluation and estimation of epidemic trajectories for SARS-CoV-2 from clinical and wastewater data in Gauteng Province, South Africa

**DOI:** 10.1371/journal.pgph.0006424

**Published:** 2026-05-07

**Authors:** Zinhle E. Mthombothi, Adrian Lison, Fiona Els, Cari van Schalkwyk, Leon Danon, Gillian Maree, Jeremy L. Bingham, Sipho Gwala, Victor Mabasa, Natasha Singh, Emmanuel Phalane, Mokgaetji Macheke, Said Rachida, Nkosenhle Ndlovu, Chenoa Sankar, Sibonginkosi Maposa, Mukhlid Yousif, Kerrigan McCarthy, Kathleen M. O’Reilly

**Affiliations:** 1 South African Centre for Epidemiological Modelling and Analysis (SACEMA), Centre for Epidemic Response and Innovation (CERI), School for Data Science and Computational Thinking, Stellenbosch University, Cape Town, South Africa; 2 Department of Biosystems Science and Engineering, ETH Zürich, Basel, Switzerland; 3 Gauteng City-Region Observatory (GCRO), a Partnership of the University of Johannesburg, the University of the Witwatersrand, the Gauteng Provincial Government and Organised Local Government in Gauteng (SALGA), Johannesburg, Gauteng, South Africa; 4 Centre for Vaccines and Immunology, National Institute for Communicable Diseases, Johannesburg, Gauteng, South Africa; 5 School of Public Health, University of Witwatersrand, Johannesburg, Gauteng, South Africa; 6 Department of Engineering Mathematics, University of Bristol, Bristol, United Kingdom; 7 Department of Virology, School of Pathology, University of Witwatersrand, Johannesburg, Gauteng, South Africa; 8 Centre for Mathematical Modelling of Infectious Diseases, Faculty of Epidemiology and Population Health, London School of Hygiene and Tropical Medicine, London, United Kingdom; SUPA71 Co., Ltd, THAILAND

## Abstract

Inferring epidemic trajectories of viral infections from wastewater data can be a useful addition to clinical-based surveillance, as it provides low cost, population-level data that includes both symptomatic and asymptomatic individuals who contribute to the sewer system. However, methods for analyzing wastewater data have been primarily applied to high-resource settings. It remains an open question to what extent epidemic dynamics can also be estimated from wastewater data in low-resource settings, where measurements are less frequent and the underlying catchment population is not clearly characterized. We used SARS-CoV-2 wastewater data from the Gauteng Province in South Africa (June 2021 to March 2022). We used the R packages EpiSewer and EpiNow2 to estimate the effective reproduction number (Rt) from wastewater data and geographically matched clinical surveillance data, respectively. The comparison between wastewater and clinical Rt showed that the observed trends are not perfectly aligned. Despite these differences, wastewater and clinical Rt estimates identified similar transmission patterns, which were similar to the trend seen directly from the recorded data. Maximum wastewater Rt of 1.38 (95% CI: 1.17–1.44) was observed in early November 2021, whilst clinical Rt was 1.18 (90% CI: 0.85–1.27) in June 2021. The change in Rt aligned with an increase or decrease in recorded cases. Our findings demonstrate that, even with limited data, estimating epidemic trajectories is feasible, providing valuable insights for informing public health recommendations. However, whenever possible, we recommend using wastewater surveillance as a complementary tool for clinical surveillance.

## Introduction

Monitoring public health is a cornerstone of effective disease management and control, particularly during outbreaks and pandemics. Traditional methods of tracking infection rates, such as clinical testing and reporting, provide valuable insights but often come with limitations, including delays in data collection, testing biases, and logistical challenges in widespread implementation. Testing in itself has some barriers which can significantly reduce testing acceptance and participation. Barriers include people being cautious to test due to financial consequences such as loss of income during the isolation period, time taken to get tested and transport costs to the test venue [1]. In recent years, the use of wastewater-based epidemiology (WBE) has emerged as a complementary and innovative approach to monitor community health, particularly in tracking infection rates of various pathogens, including viruses like SARS-CoV-2, the virus responsible for COVID-19 [2]. WBE is the practice of analysing pathogens and chemicals in wastewater to gain insights into the health status of a population [3]. The rationale behind WBE is that many pathogens, drugs, and other biomarkers of health conditions are excreted by humans through feces and urine and can be detected in wastewater (WW). By sampling and analysing sewage (effluent) from communities, it is possible to monitor trends in the presence of certain conditions or infections within a community.

The WBE approach has three advantages over traditional surveillance methods. Firstly, WBE offers a more comprehensive and less biased snapshot of a community’s health. Traditional surveillance methods rely on individuals seeking medical attention and getting tested, which can result in underreporting, especially among asymptomatic or mild cases, and in settings with limited health-care access. In contrast, WW sampling captures data from everyone who contributes to the sewer system, providing a more inclusive measure of infection rates. Secondly, wastewater monitoring can detect outbreaks at an early stage, often before clinical cases are reported [4]. Infected individuals may shed pathogens in their waste even before symptoms appear; consequently, wastewater data can serve as an early warning system and as a way to monitor genomic variants through sequencing [5]. This early detection capability allows for more proactive public health responses, and if linked to interventions, can potentially curb the spread of disease before it becomes widespread. For instance, during the COVID-19 pandemic, WBE was instrumental in identifying hotspots of infection, sometimes weeks before increases in clinical testing confirmed rising case numbers [ 4,6,7]. Thirdly, WBE is scalable, making it an attractive option for continuous monitoring over large areas. Unlike mass testing, which requires substantial resources and can be logistically challenging, wastewater sampling can be conducted at key points in a sewer system, such as treatment plants, to capture data from entire communities from one sample. This scalability is particularly advantageous in resource-limited settings where extensive clinical testing may not be feasible.

Despite its advantages, using WW data to estimate infection rates comes with challenges that need consideration. The interpretation of WBE data is complex, as the concentration of pathogens may not directly track numbers of infections, as it may be influenced by factors such as population size, wastewater flow rates, deterioration of nucleic acids, environmental conditions, and pathogen-specific stability in wastewater [8]. Additionally, quantifying the exact number of infections from wastewater data requires sophisticated modelling and a grounded understanding of the local context [9].

A key metric used to understand the epidemic potential of a pathogen is the effective reproduction number (Rt), the average number of secondary infections per infection within a population at a specified time. If Rt > 1, an outbreak is likely and consistent with an increasing number of infections, while values below 1 are consistent with a decreasing trend in infections. Statistical methods have been developed to estimate Rt from reported case data, while accounting for pathogen-specific properties such as the time between symptom onset of connected cases (known as the serial interval), time between infections (known as the generation interval), and delays in reporting [10]. The estimation of Rt within specific communities was a key metric used by public health stakeholders during the COVID-19 pandemic to forecast the suitability of hospital bed capacity and evaluate the effectiveness of interventions [11,12].

Several aspects of WW surveillance require consideration within a modelling framework. The measured concentrations of viral RNA in WW provide a population-level indicator of pathogen shedding, which is comparable to prevalence, whereas Rt estimation requires a measure of new infections within a specified time. Interpretation of pathogen measurements from WW samples has additional challenges, such as the complexity of varying dilution (often accounted for by measuring the WW flow measurements), uncertainty in the size of the catchment population, and infrequent sampling. These factors can impact the accuracy and precision of Rt estimates. Several research groups have extended statistical methods for estimating Rt from clinical data to consider the usage of WW data [13–15]. A recent study comparing eight models that estimate Rt from wastewater data found a high degree of similarity across all models despite the models requiring different model parameters and approaches [16].

Although these models have been tested in high-resource settings, there is a need for such models in low-resource settings where data collection may be limited. Therefore, this study aimed to examine how modelling frameworks for Rt estimation are affected by data restrictions that are common in low-resource settings. We then apply the model to clinical and WW data from South Africa.

## Materials and methods

### Ethics statement

This analysis of secondary data was approved by the LSHTM ethics committee (Ref 28269, 18th January 2023). Ethical approval for wastewater data collection and analysis was obtained from the University of Witwatersrand Human Research Ethics Committee (#M220904).

### Study setting and data sources

To examine how modelling frameworks for Rt estimation are affected by data limitations usually experienced in low-resource settings, we used WW data of SARS-CoV-2 viral concentrations as reported by The National Institute for Communicable Diseases (NICD) of South Africa [17]. Wastewater samples in this surveillance programme were collected mainly using grab sampling, rather than composite sampling which is more commonly used in high-income countries. Across the country data were collected from 15 wastewater treatment plants (WWTPs): Gauteng, Kwa-Zulu Natal, Western Cape, Eastern Cape, and Free State.

One-litre influent grab samples were collected during morning peak hours (between 7am and 11am) and transported to the NICD at <5 °C. Flow rate and volume data were not recorded by the sample collection teams. RNA extraction was done the same day the sample arrived in the laboratory, and laboratory testing was done as previously described [ 4]. In the laboratory, 200 mL of each sample was centrifuged at 4650 × g (4°C, 30 min), and 70 mL of the supernatant was further concentrated to 1 mL using a Centricon Plus-70 filter (3500 × g, 15 min). A 140 µL aliquot was spiked with 5 µL of the Allplex 2019-nCoV internal control and RNA extracted using the QIAamp Viral RNA Mini Kit (Qiagen), eluted in 50 µL AVE buffer, and tested or stored at −20 °C as an additional sample for future testing of other pathogens if necessary.

The RNA EDX Standard (Bio-Rad) was double extracted, eluted in 50 µL AVE buffer, and serially diluted (1:4096–1:4; 2.34–2400 genome copies). Triplicate 8.5 µL aliquots of each dilution were stored at −20 °C for concurrent use with samples. Viral RNA was detected and quantified using TaqMan-based RT-PCR on the Applied Biosystems 7500 Real-Time PCR System.

At the initiation of this analysis, we determined that a minimum dataset was required for estimation of Rt in time: routine collection of SARS-CoV-2 gene copies at a minimum of weekly intervals, corresponding wastewater flow data from the WWTPs, and the estimated size of the population catchment areas to enable accurate comparison to clinical cases. Of the 15 WWTPs included in the national surveillance dataset, only two met the criteria required for the primary modelling framework. Most catchments did not have flow data available from the treatment plants, and in some cases clinical case data were not available for the corresponding period of analysis. Because the modelling framework requires both wastewater flow measurements and case data corresponding to the specific WWTP catchment area, the analysis first focused on two WWTPs in Gauteng province (Site D and Site O), for which both flow measurements and catchment-level clinical case data were available. To examine how the modelling framework performs under more typical data constraints common in low-income settings, we also conducted the analysis for two additional WWTPs (Site G and Site R) that did not have flow measurements available and for which only district-level case data could be used.

The catchment areas of Site D and Site O WWTPs differ in population size and socio-economic status. Site D is a residential area with established infrastructure with a population of about 245,900, with formal housing in the west and central areas. Whilst the east is home to the metropolitan central business district, informal housing is found in the far west. Site O is mostly residential with a population of approximately 900,000; there is ongoing industrial and manufacturing activity to the northeast [18]. Detailed descriptions of the study sites are available in Maree et al. (2025) [18]. Site G serves large residential and commercial areas in the southern parts of the metropolitan municipality, with an estimated catchment population of over one million residents. Site R receives wastewater from northern Pretoria and surrounding urban areas, serving a catchment population of several hundred thousand residents.

Clinical SARS-CoV-2 case data were accessed for research purposes on 21 August 2023. Our analysis is based on data collected between June 2021 and March 2022, a period when SARS-CoV-2 concentration was recorded weekly across all catchment areas. Confirmed clinical cases data from within the catchment areas were reported weekly, and daily flow data were recorded for the selected study Sites D and O. For Site G and R, district-level case data were recorded weekly and there was no flow data reported.

Clinical cases of COVID-19 within the catchment areas consist of cases reported to public and private laboratories and returning a positive SARS-CoV-2 test. During the time period under consideration, South Africa did not have rapid home-based tests; COVID-19 testing was conducted primarily at designated testing facilities. Reporting of cases remains legally mandated, with included geocoding that enabled alignment within WWTP catchment areas. However, testing behaviour has changed since the acute phase of the COVID-19 pandemic, with fewer people getting tested compared to during the pandemic, resulting in a decline in reported cases [19].

Site O had very limited data coverage with only four data points available during the analysis period. We included this site as it was one of the two sites for which both wastewater, flow and clinical case data were available.

**Fig 1:**
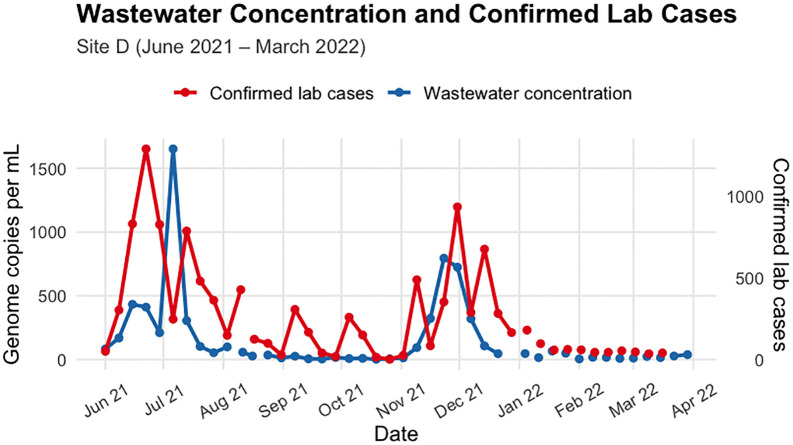
Catchment specific laboratory-confirmed weekly cases and SARS-CoV-2 concentrations in wastewater at Site D, June 2021 – March 2022. WW data were collected approximately weekly.

**Fig 2:**
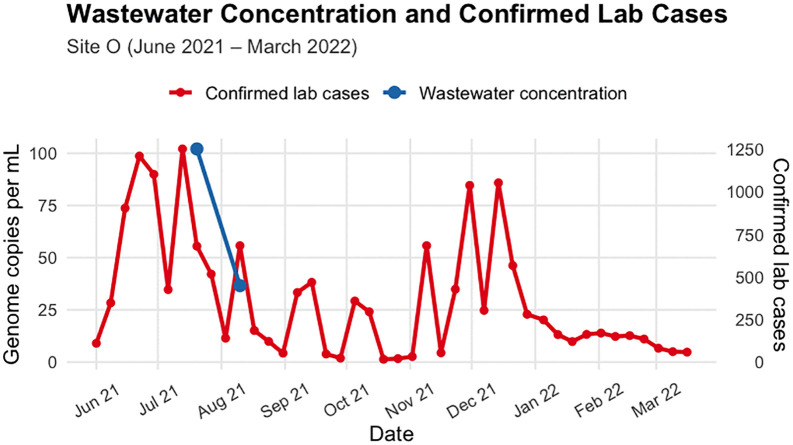
Catchment specific laboratory-confirmed weekly cases and SARS-CoV-2 concentrations in wastewater at Site O, June 2021 – March 2022. WW data were collected approximately weekly.

Figs 1 and 2 show trends in catchment specific lab-confirmed COVID-19 cases alongside wastewater SARS-CoV-2 concentrations for Site D and Site O, respectively. We observed peaks in June and December 2021; however, Fig 2 (wastewater concentration) highlights a period of missing data for Site O, which was mentioned above, but in this plot, we only show data for the analysis period.

Sites G and R were chosen to highlight areas with limited data available, for the analysis of these sites we will use wastewater data, district-level case data and a fixed flow data as we do not have any flow data available.

**Fig 3:**
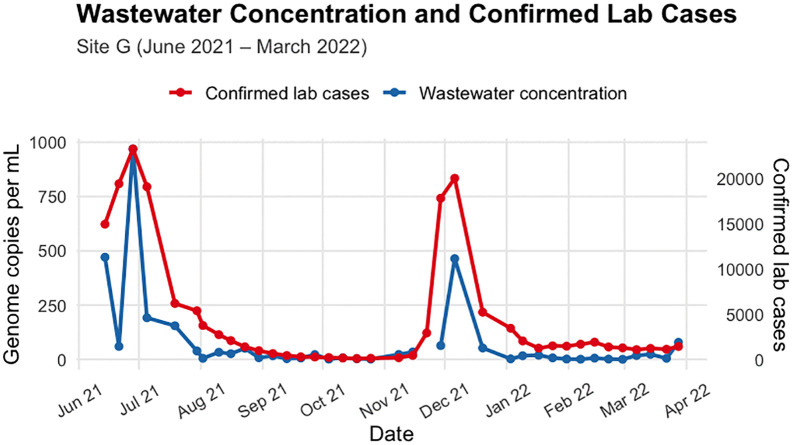
District-level laboratory-confirmed weekly cases and SARS-CoV-2 concentrations in wastewater at Site G, June 2021 – March 2022. WW data were collected approximately weekly.

**Fig 4:**
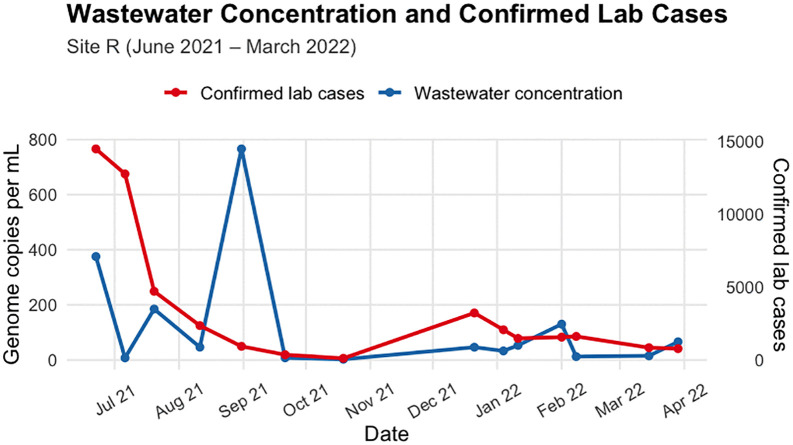
District-level laboratory-confirmed weekly cases and SARS-CoV-2 concentrations in wastewater at Site R, June 2021 – March 2022. WW data were collected approximately weekly.

Site G has two apparent peaks for both clinical cases and wastewater concentrations occurring in early July and early December (Fig 3). Whilst Site R has one big peak in wastewater concentration towards the end of August, and a smaller peak in clinical cases towards end of December and another small peak in wastewater concentration around early February (Fig 4).

### The effective reproduction number, Rt

Empirical estimates of Rt can be derived from the number of infections reported in time and the generation interval (i.e., the time between the infection of a primary case and a secondary case). Typically, statistical distributions are used to reflect variability in estimates of the generation interval. The relationship between these three components can be defined via the renewal equation:


$$E\left[I_{t}\right]=\;R_{t}\;\sum_{s=\text{1}}^{G}g_{s}I_{t-s}\;|\;t>G$$ (1)

where $I_{t-s}\;$ is the number of realized infections that occurred s days before t, and $g=\;g_{\text{1}},\;g_{\text{2}},\ldots ,\;g_{G}$ is the discrete generation interval distribution with maximum generation time G.

Therefore, the expected number of infections ($E[I_{t}]$) on day t, depend on the number of infections that occurred on previous days, weighted by the probability that those earlier cases generated a new infection s days later. The weights $\;g_{s}\;$are derived from the generation interval distribution, which describes how likely transmission is to occur at each possible lag time.

Typically, Rt has been estimated from reported cases using the above renewal equation, including methods that account for under-ascertainment and censoring, which are inherent in disease surveillance.

### Estimation of Rt from wastewater data

Several methods for estimating Rt from wastewater data have been recently developed. The core principle of this approach, first developed in Huisman et al. [10], is to use measured pathogen concentrations in wastewater samples to infer the number of infections in the wastewater catchment over time, which in turn allows the estimation of Rt using the renewal equation described above. For example, the method proposed by Huisman et al. uses a deconvolution approach to estimate the infection incidence from the daily wastewater load (total number of gene copies shed by individuals within the catchment on a given day), which can be obtained by multiplying the concentration (in gene copies per ml) by the daily flow (in volume units, such as megalitres, with appropriate scaling).

As infected individuals may shed genetic material of the pathogen over a longer period of time with varying intensity, a shedding load distribution is used to deconvolve the load time series into an infection time series, assuming that


$$E\left[I_{t}\right]=\;\sum_{s=\text{0}}^{S}\mu^{load}\overset{shed}{\underset{s}{\;\tau }}\;I_{t-s}\;$$ (2)

where $E[l_{t}]$ is the expected load in the catchment on day t, $\mu^{load}$ is the average number of detectable gene copies per infected individual and $\tau^{shed}=(\overset{shed}{\underset{\text{1}}{\tau }},\;\overset{shed}{\underset{\text{2}}{\tau }},\;...,\;\overset{shed}{\underset{S}{\tau }})$ a discrete shedding load distribution with shedding up to S days after infection. To model the shedding load distribution, a positive continuous distribution is often used, e.g., log-normal distribution with a given mean and standard deviation and discretized into daily bins. Since the average shedding load per individual $\mu^{load}$ depends on various parameters, including site- and lab-specific factors, it is typically obtained by calibration to observed case numbers or by making simplifying assumptions. For example, one could assume that the smallest measured load corresponds to that of a single infection [10]. However, sensitivity analyses have shown that, within realistic bounds, the effect of this scaling factor on trend estimates, including Rt estimates, is limited, making misspecification of $\mu^{load}$ a less serious issue.

One limitation of deconvolution-based approaches is that they require the load trajectory to be sufficiently smoothed and the measurements on unsampled days to be interpolated. Furthermore, it is challenging to propagate uncertainty from smoothing and interpolation to the subsequent deconvolution and Rt estimation steps. These limitations are especially critical in settings with sparse and potentially noisy measurements, such as in the Gauteng Province. Therefore, we here use an alternative approach proposed in [20,21] and implemented in the R package “EpiSewer” [15]. In this approach, a hierarchical Bayesian model is used to jointly infer measurement errors, wastewater loads, infections, and Rt within a single model. This renders additional smoothing and interpolation of measurements obsolete and provides a more comprehensive quantification of Rt uncertainty. The model consists of five submodules (measurement, sampling, sewage, shedding, and infection). In the following, we describe the specification of each module used in our analysis.

In the measurement module, we modeled unreplicated qPCR measurements, assuming that measurement errors are Gamma distributed with a constant coefficient of variation ν. We used a truncated normal prior with a mean of 0 and a standard deviation of 1 for ν. The limit of detection for samples using the NICD procedure is 1.49 per genome copies/ml.

In the sampling module, we assumed that samples were representative of the daily wastewater concentration in the catchment but accounted for potential outlier concentrations using an extreme value distribution model provided in EpiSewer.

In the sewage module, we used the daily wastewater flow into the wastewater treatment plant to adjust the observed RNA concentration for dilution from rainfall and other factors, thereby providing an estimate of the viral load in the catchment. Wastewater flow from the residents to the wastewater treatment plant can potentially take longer than 24 hours, and a residence time distribution can be specified to account for this delay. However, in our application, we made the simplifying assumption that all sewage reaches the sampling site within 24 hours. Viral degradation during in-sewer transport and storage before processing may reduce the detectable SARS-CoV-2 RNA concentrations in wastewater samples. However, previous work has shown that transmission indicators such as the effective reproduction number (Rt) are generally robust to reductions in detectable concentrations, provided that degradation does not vary systematically over time [13,21]. Environmental and logistical conditions at our sampling sites were relatively stable over the monitored period, and therefore we do not expect sample degradation to have substantially affected our Rt estimates.

In the shedding module, we calibrated the average shedding load per case such that the estimated number of infections was on the same order of magnitude as the number of reported cases. We accounted for variation in shedding intensity between individuals, assuming a coefficient of variation of 1. For the time distribution of shedding since the date of infection, we used a discretized Gamma distribution with uncertain mean and coefficient of variation as specified in [20]. The priors for this shedding load distribution are based on estimates from [22] and range from 9.16-15.69 days for the mean and between 0.53-0.87 days for the standard deviation.

In the infections module, we smoothed the effective reproduction number over time via a Gaussian process model, using the default smoothing priors of EpiSewer. We modeled the number of new infections as negative-binomially distributed with an overdispersion parameter of 10%. For the generation time, we assumed a Gamma distribution with a mean of 4.7 days and a standard deviation of 2.9 days [23].

Estimates of Rt and other parameters were obtained via Markov Chain Monte Carlo (MCMC) sampling using the No-U-Turn Sampler (NUTS) in cmdstan v2.34.1 with 500 warm-up and 1000 sampling iterations. We summarized posterior samples using the median and 50% and 95% credible intervals. The robustness of Rt estimates against measurement noise, outliers, and various forms of model misspecification was demonstrated in Lison *et al.* [21]

Wastewater concentrations data and flow data are required to estimate Rt from EpiSewer. EpiSewer can work with missing concentration data and requires flow data for the days with concentration measurements. EpiSewer requires the units for concentration data to be gene copies per mL and flow data to be mL per day.

The different models are summarised in Table 1, highlighting the input parameters we used as well as the model outputs. The input parameters mentioned are those required by the model; there are other optional input parameters.

**Table 1:** Model summary with respective input and output parameters.

Model	Purpose of model	Input parameters	Output parameters
EpiSewer	Estimate Rt using wastewater	• Wastewater concentration measurements • Flow data	• Rt estimates • Infection estimates • Wastewater concentration
EpiNow2	Estimate Rt using clinical data	• Time series of confirmed cases • Reporting delay from symptom onset to case reporting • Generation time distribution and incubation period	• Time series of R • Daily new reports • Daily new infections • Short-term forecasts

### Rt estimation from clinical case data

The EpiNow2 R package estimates time-varying reproduction number (Rt), epidemic growth rates, and doubling time from clinical data. EpiSewer and EpiNow2 use the same general approach (generative modelling, hierarchical Bayesian modeling). EpiNow2 propagates uncertainty from all input sources—such as delays in reporting and uncertain incubation periods—into the final Rt estimates. This method allows for more reliable and interpretable estimates of transmission dynamics by inferring infection dates from reported case counts. It also performs short-term forecasting using the same generative process approach used for estimation [24].

The expected number of new infections at time t is given by:


$$I_{t}=\;R_{t}\;\sum_{\tau =\text{1}}^{g_{max}}g(\tau |\theta_{g})\;I_{t-\tau }$$ (3)

where $g_{t}\;=\;g(\tau |\theta_{g})$ is the discretised distribution of generation times with parameters $\theta_{g}\;$and maximum $g_{max}$ [25].

The incubation period was modeled using a Gamma distribution with shape parameter 8.5, scale parameter 0.5 and maximum of 8 days. The truncated mean incubation period was about 4.25 days [26]. For reporting delays, we used a log-normal distribution with mean 2 and standard deviation 1, truncated at 10 days. The generation time distribution is the same one we used for the wastewater analysis.

All analyses were computed in R (version 4.4.3), including the implementation of EpiNow2 and EpiSewer for estimating time-varying reproduction numbers.

This analysis of secondary data was approved by the LSHTM ethics committee (Ref 28269, 18th January 2023). Ethical approval for wastewater data collection and analysis was obtained from the University of Witwatersrand Human Research Ethics Committee (#M220904)

## Results

### Rt estimation in Gauteng, South Africa

**Fig 5:**
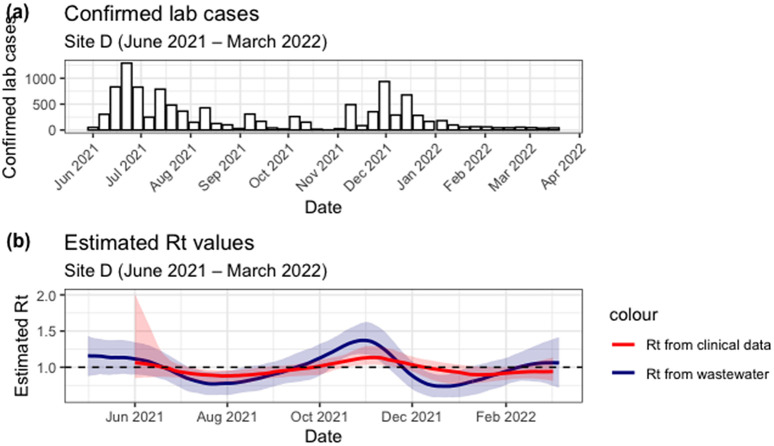
(a) Lab confirmed COVID-19 cases for Site D. (b) shows estimated Rt from clinical cases (red line with shaded 90% credible interval) and from wastewater (blue line with shaded 95% credible interval).

**Fig 6:**
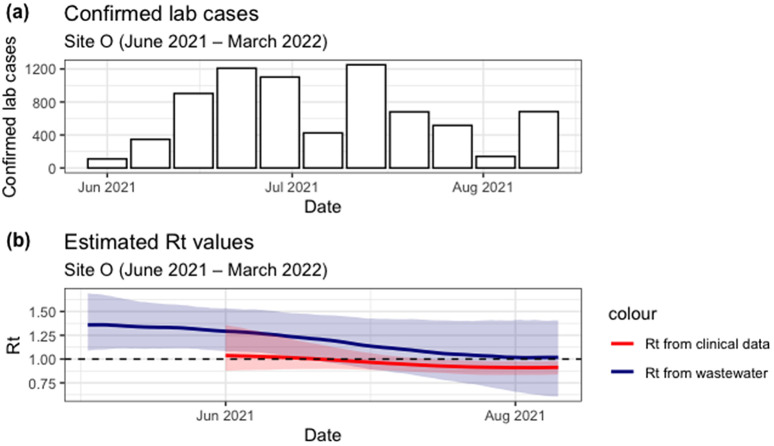
(a) Lab confirmed COVID-19 cases for Site O between June and August 2021. (b) Estimated Rt from clinical cases (red line with shaded 90% credible interval) and from wastewater (blue line with shaded 95% credible interval) for Site O.

Between March 2021 and June 2022, South Africa experienced two waves, where there was an increase in the number of new COVID-19 cases. The first increase was noted around June-July 2021, and the second one around November 2021. As can be seen in Figs 5(a) and 6(a) below.

Using EpiSewer and EpiNow2, we estimated Rt from wastewater and clinical data, respectively.

Fig 5A shows the number of lab-confirmed cases for Site D. Fig 5B shows a comparison of Rt estimates derived from wastewater and case data for Site D WWTP. The Rt remains relatively stable through time. Wastewater Rt has large uncertainty bands, whilst clinical Rt is less variable. Wastewater Rt estimates show three peaks in June 2021, November 2021, and March 2022, whilst clinical Rt estimates show one peak in November 2021. We note that around August 2021, and again around January 2022, wastewater Rt estimates seem to rise earlier than clinical Rt estimates. Despite these differences, both Rt estimates identify similar overall transmission trends, with the highest Rt value of 1.18 (90% CI: 0.85 - 1.27) from clinical data in June 2021 and 1.37 (95% CI: 1.17 - 1.44) from wastewater data recorded in early November 2021. The predicted Rt trend is similar to the trend of recorded cases. At the beginning of the simulation, Rt from clinical data was slightly greater than one, indicating an expected increase in the number of cases. Rt from wastewater was clearly above one, indicating that an increase in cases should be expected. Furthermore, from the data we see that cases increased, reaching their peak in June 2021. Thereafter, Rt dropped to less than one towards the end of June, and by the end of July, we saw a decrease in the number of cases recorded. There is a delay from when the Rt from wastewater goes below one to when we see a decrease in the number of cases, which reflects the delays between infection, symptom onset, and reporting. Wastewater may therefore provide an earlier signal for change in transmission dynamics.

Fig 6A shows the number of lab confirmed cases for Site O for the duration when WW data was available. Site O only had complete data for clinical cases and flow, and very sparse data for wastewater. Due to insufficient data for wastewater, the WW Rt was estimated for the period between June and August 2021. However, the comparison was done for the period where both wastewater and clinical Rt are defined, Fig 6B. WW Rt shows that Rt was declining between June and August 2021, but stays above one, which indicates that we are still expecting a rise in cases. The highest WW Rt value of 1.37 (95% CI: 1.10 - 1.68) was observed in early May 2021. Clinical Rt starts above one, and quickly goes below one, which is more representative of the data, as cases were initially rising but went down in early July.

**Fig 7:**
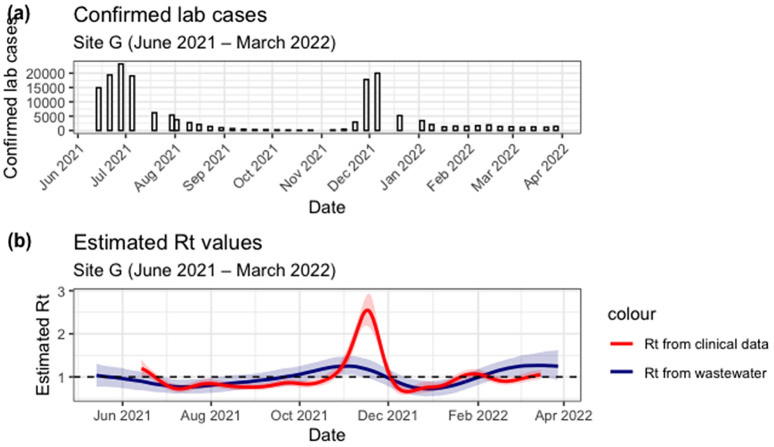
(A) Lab confirmed COVID-19 cases for the district associated with Site G between June 2021 and March 2022. (B) Estimated Rt from clinical cases (red line with shaded 90% credible interval) and from wastewater (blue line with shaded 95% credible interval) for Site G were generated assuming constant flow and using district-level case data.

Fig 7A shows the number of lab confirmed cases for the district associated with Site G between June 2021 and March 2022. Fig 7B shows Rt estimated under a reduced data setting, we assumed constant flow and we also used district-level clinical cases. Wastewater-derived Rt has larger uncertainty bands compared to clinical Rt, yet clinical Rt has the biggest peak. Wastewater Rt has two peaks, initial peak in early October 2021 then another one in early February 2022, whilst clinical Rt has one big peak mid-November 2021. Maximum WW Rt is 1.25 (95% CI: 1.05 - 1.48) observed in early November 2021. Clinical Rt recorded the highest value mid-November 2021 with a value of 2.54 (90% CI: 2.17 – 2.93). Though the peak for clinical Rt is higher than that of WW Rt, the transmission trend is similar, with WW Rt going above 1 before clinical Rt.

**Fig 8:**
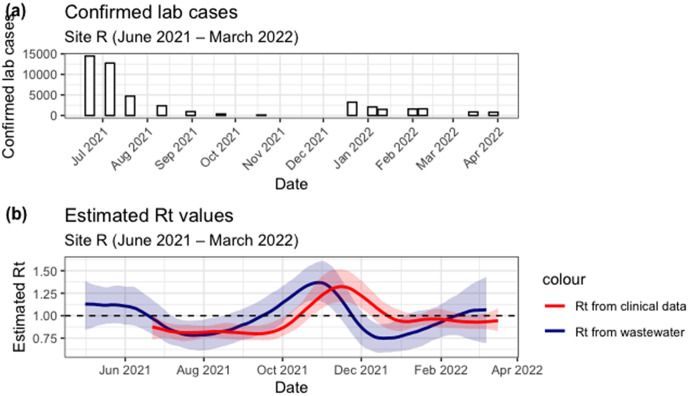
(A) Lab confirmed COVID-19 cases for the district corresponding to Site R between June 2021 and March 2022. (B) Estimated Rt from clinical cases (red line with shaded 90% credible interval) and from wastewater (blue line with shaded 95% credible interval) for Site R were generated using constant flow and district-level case data.

Fig 8A shows Lab confirmed COVID-19 cases for the district corresponding to Site R between June 2021 and March 2022. Smaller case count is reported with a clear decline after July 2021. Both estimated Rt trends have one peak which also corresponds to the max Rt values Fig 8B. WW Rt was the highest around end of October 2021, with value of 1.37 (95% CI: 1.17 – 1.60) and clinical Rt around mid-November 2021 with a value of 1.32 (90% CI: 1.15 – 1.52). The timing of growth and decline is consistent between the two estimates.

### Exploration of metadata required for accurate estimation of Rt

The data from Zurich, Switzerland, which was used to validate EpiSewer, has daily recorded wastewater concentrations, daily flow, and daily case data. In contrast to the South African data, which has wastewater concentrations sampled weekly and clinical cases recorded weekly. To validate the use of EpiSewer in settings with less frequent data collection we performed a data reduction exercise where we reduced the data from Zurich to resemble the South African data. First, we remove daily wastewater concentrations and only leave Tuesday entries, as to only have one entry per week. In the next exercise, we removed and replaced the flow data with a constant flow rate (average flow), motivated by the missing flow data in many low-resource settings.

To quantify the impact of estimating Rt using data with weekly sampling instead of daily sampling, we calculated the mean absolute error (MAE). The mean absolute error measures the average absolute deviation between the Rt estimates obtained from the full dataset and those obtained from the reduced dataset. We consider an MAE below 0.1 to indicate an epidemiologically negligible difference. The MAE between daily sampling (actual data) and weekly sampling (reduced data) was 0.038, while the MAE for daily flow (actual data) vs constant flow (reduced data) was 0.062. Since both MAEs are below 0.1, this indicates that the reduced data had minimal effects on Rt estimation, suggesting that using limited data may still provide reliable Rt estimates. Based on the MAE results, we infer that the South African data is sufficient to produce meaningful Rt estimates.

## Discussion

This study aimed to determine to what extent epidemic dynamics can be estimated from wastewater in a low-resource setting, where sampling typically occurs once a week, the catchment population may be poorly defined, and flow data is limited or non-existent, and to examine how data limitations in such settings affect Rt estimation. From our data reduction exercise, we found the mean absolute error between daily sampling and weekly sampling to be 0.038, while the MAE for daily flow vs constant flow was 0.062, which are both below 0.1, corresponding to an epidemiologically small difference. These analyses indicate that minimal information is lost from using the reduced dataset; by extrapolation, the South African dataset is therefore likely sufficient to provide reliable Rt estimates that may be used to inform public health decisions. This finding is important for low-resource settings as weekly sampling is far more practicable, reduces the workload and cost with minimal impact on Rt estimation. However, weekly wastewater sampling may still limit the agility of early epidemic detection and should be considered in specific circumstances such as the early stages of an emerging pandemic.

Regarding frequency of testing, our findings contrast with a study from Singapore, which suggested that models using weekly sampling were hardly informative, with Rt estimates being noisy and imprecise when compared to models with daily sampling [27]. In this data-rich environment, the authors evaluated the ability of data streams (total cases, hospitalisations, clinical data, and wastewater data) to forecast hospitalisations. For the entire period, hospitalisation data were the best predictor for hospitalisations, and for December 2022 - May 2023, during which there was a reduction in case reporting, the inclusion of wastewater data reduced model error. The authors comment that wastewater data enhances the reliability of Rt estimates and may be especially important if there are substantial changes in case ascertainment. During real-time monitoring, weekly sampling might also lead to a delay in characterising transmission trends since we only update the data once a week. These concerns are also supported by a previous study that reported that to reliably estimate Rt from wastewater samples had to be collected at least three times a week, with more frequent sampling suggested for real-time monitoring [13]. Also, for Rt estimates to be both reliable and informative, they had to be sampled at least twice a week – these models performed similarly to models with daily sampling [27]. On the other hand, CDC recommends sampling once a week if the goal is to screen for the presence of SARS-CoV-2 over time [28]. Use and sampling strategies of WW therefore, need to consider practicability, and the need for accuracy of epidemiological metrics, especially where there are concerns regarding case ascertainment.

When comparing wastewater and clinical Rt estimates, we found that the observed trends are not perfectly aligned. This difference may arise through sensitivity in either wastewater or clinical data to short-term changes in transmission dynamics, changes in case ascertainment in clinical data, or unexplained noise in WW data. Regardless, in our observations, WW and clinical Rt estimates predicted similar transmission trends. In some periods (e.g., August and January-February), the WW Rt increased faster than the clinical Rt, suggesting that WW-derived Rt signals a change in transmission dynamics. Another study suggested that WW SARS-CoV-2 RNA concentrations reflect a change in the number of new cases rather than reflecting the dynamics of prevalent infections [29]. Therefore, higher than expected WW SARS-CoV-2 RNA concentrations can serve as an early warning tool for emerging outbreaks [29]. Occasional spikes in SARS-CoV-2 concentrations may also be due to sampling noise, and it is hard to distinguish the true signal from noise based on WW data alone [30]. These challenges are dealt with within the Rt estimation methods used in our paper, as high concentrations are modelled as explicit measurement noise. Despite this, it remains important to use other data sources (i.e., clinical or hospital data) to support observations and to strengthen the decisions made.

Insight into the validity of wastewater-derived Rt estimates is limited by weakness in clinical surveillance, namely that laboratory-based testing underestimates disease burden [9]. In our dataset, it was previously demonstrated that the two sewersheds O and D have different socioeconomic conditions, different population sizes, and similar measured concentrations of SARS-CoV-2, yet they had similar absolute weekly SARS-CoV-2 case numbers. The equivalent SARS-CoV-2 concentrations in the two sewersheds suggested under-reporting in clinical cases in the less privileged sewershed, likely due to limited access to health care and other socio-economic considerations [18]. Therefore, our clinically derived Rt estimates may not represent the true burden of disease.

Our results show that after Rt decreases to below one, we then see a decrease in clinical cases. These findings support the use of Rt estimates for planning purposes, as Rt can inform when to expect an increase or decrease in cases, which will inform how to better assign resources according to need. Another study showed that wastewater outperformed clinical case data with respect to the timing and shape of the peak incidence, whilst case numbers were a better indicator for incidence decline [31]. A study in India found that SARS-CoV-2 RNA concentrations in WW could be used to estimate the number of infected individuals. Their model predicted that the reported clinical cases were 12–14 times lower, indicating substantial underreporting [32]. Thus, demonstrating that WW surveillance can capture missed infections whether due to limited clinical testing resources or many cases are asymptomatic or not reported. The best practice is to use WW data with clinical case data as they complement each other.

Analyses were conducted for Sites G and R used district-level case data and assumed constant flow. Even under these reduced-data conditions, wastewater Rt still captured major transmission trends, identify increasing and decreasing transmission periods, which were consistent with those inferred from clinical data. The results from the supplementary analysis, which is a scenario more common in low-income settings. The wastewater-derived Rt estimates remained informative even when flow data and catchment-level case data was unavailable.

Wastewater-based epidemiology has increasingly been recognised as a valuable surveillance tool, particularly in settings with limited or inconsistent clinical testing capacity. Several studies demonstrated the feasibility of monitoring SARS-CoV-2 transmission through wastewater in different settings, including in African contexts where surveillance systems are often inadequate due to limited resources [33]. It was also shown that even when grab sampling was used or sampling frequency was limited, wastewater surveillance can still provide useful population level indicators for infection dynamics [34,35]. In addition, recent modelling work has shown that wastewater data can be used to estimate epidemiological indicators such as the reproductive number and track changes in infection [13]. Our findings support these observations and suggest that wastewater-derived Rt estimates can provide meaningful insights into transmission dynamics even when using limited data as typically found in low-and-middle income countries. This is particularly important in the South African context, where declining clinical testing and uneven access to healthcare may limit the reliability of clinical surveillance.

SARS-CoV-2 RNA was detected even in very low COVID-19 incidence periods [36]. Wastewater data has also been able to identify new variants before clinical data [36,37]. Proving that adding WW surveillance will strengthen sensitivity of event detection for outbreak and monitoring of endemic disease trends [37]. The NICD developed a WW surveillance dashboard which went live in 2021. The dashboard now includes both WW and clinical case surveillance. The real-time and interactive dashboards for wastewater-integrated surveillance can support timely and informed public health decision making [38]. As testing has decreased the dashboard helps monitor trends in WW. The dashboard can be adapted for other pathogens and be used to monitor transmission dynamics and inform public health decisions.

This modelling framework is not yet routinely implemented within the South African public health system, although wastewater surveillance is increasingly being used to monitor different pathogens [18,38]. Our findings show that wastewater-derived Rt estimates can provide useful insights into transmission dynamics, especially in areas where clinical testing is limited. However, wastewater data should not be used in isolation. From a public health perspective, relying on wastewater data alone could lead to over or underestimation of transmission and potential misallocation of healthcare resources. Wastewater-based Rt estimates should therefore be interpreted alongside clinical and other surveillance data [31]. Overall, this study highlights the value of wastewater-based Rt estimation as a complementary tool for monitoring transmission in resource-constrained settings.

## Conclusion

Our findings demonstrate that Rt estimated from WW data - even when sampled weekly, as generally found in low-resource settings - can be informative and complement estimates from clinical data alone. Wastewater Rt and clinical Rt appear to have a similar trend though not perfectly aligned. These minor discrepancies highlights that the two methods complement each other and capture different aspects of transmission dynamics. Importantly, our results suggest that in settings where clinical surveillance is non-existent or limited, even if sampling occurs less frequently than recommended, WW Rt may be used to monitor changes in SARS-CoV-2 transmission patterns and inform public health recommendations. However, less frequent sampling may miss short-term changes in data, which may potentially delay the detection of emerging outbreaks. Availability of flow data is important, especially if there are large fluctuations in flow as these would affect the accuracy of Rt. In conclusion, our findings highlight the potential value of wastewater-based Rt estimation as a complementary surveillance tool for monitoring transmission dynamics in low-resource settings.
